# Cytokine and immune cell interaction in immune-inflammatory response during crisis event in sickle cell disease

**DOI:** 10.4314/ahs.v25i1.36

**Published:** 2025-03

**Authors:** Suprava Patel, Saurav Nayak, Diksha Chandrakar, Preetam N Wasnik, Tushar B Jagzape, Eli Mohapatra, Rachita Nanda, Seema Shah

**Affiliations:** 1 Department of Biochemistry, AIIMS, Raipur, Chhattisgarh, India; 2 Department of Biochemistry, AIIMS, Bhubaneswar, Odisha, India; 3 Department of Medicine, AIIMS, Raipur, Chhattisgarh, India; 4 Department of Pediatrics, AIIMS, Raipur, Chhattisgarh, India

**Keywords:** TNFα, IFNγ, IL10

## Abstract

**Background:**

The present study focused on evaluating serum cytokines in SCD cases and understanding these mediators' interplay with immune cells and their impact on disease severity score(DSscore).

**Methods:**

The study population of eighty-eight participants comprised twenty-one SCD cases with DSscore>5, thirty-seven SCD cases with DSscore≤5, and thirty control group. Blood samples were analyzed for T cell markers by flowcytometry and serum IL10, TNFα, IFNγ, and glutathione.

**Results:**

Serum IFNγ and IL10 levels were significantly higher in cases than control(p<0.05). The median(IQR) TNFα, 100.51(66.8) was higher in cases DSscore>5 than those with DSscore≤5(p=0.04). Both cases showed a significant rise in IFNγ compared to the control group(p<0.05). Similarly, median(IQR) IL10, 7.9(13.3) was elevated in cases DSscore>5(p=0.001) than control group. IL10 recorded a significant linear relationship with Tc-exhausted (CD8+CD279+) cells(r= 0.327, p=0.012) and Th-exhausted (CD4+CD279+) cells(r=0.265, p=0.045). A linear association(r=0.292, p=0.026) was observed between IFNγ and Tc Naive/Effector cell ratio (CD8+NECR). Similarly, TNFα was positively associated with the total T cell Naive/Effector cell ratio (TNECR) (r=0.307, p=0.019). Serum glutathione levels correlatedh exhausted (CD4+CD279+) cell (r=0.393, p=0.002) and IL10 (r=0.589, p<0.001). Conclusion: Cytokine profiling in SCD patients might provide insight into the underlying clinical course and vulnerability toward crisis events.

## Background

Sickle cell disease (SCD) is the most prevalent hemoglobinopathy in Central India. Although the homozygous (HbSS) form of the point mutation in the sixth codon of the β-globin gene is a prototype for the monogenic Mendelian disorder, it is known for its extreme clinical heterogeneity in individuals with it^1^. As previously thought, it is no longer a disease of sub-Saharan, the Middle East, or India. Migration pattern has enabled the distribution of this gene to various part of Europe and the United States as well. The reported incidence of sickle cell trait (SCT) in the United States in 2010 was 15.5 per 1000 newborns, 73.1 among Black and 6.9 among Hispanic newborns^2^. In India, this genetic disorder is more widespread among the tribal population, where every 1 of the 86 births has at least one allele of the sickle cell hemoglobin (HbS) gene^3^.

Crisis events associated with symptomatic anemia and pain are the major cause of hospitalization and impaired quality of life. Besides, bone marrow failure causing acute aplastic crisis, notably due to Parvovirus B19 infection, could trigger severe anemia and require blood transfusion^4^. Infections such as bacterial sepsis or malaria could trigger acute chest syndrome resulting in mortality. Various complications involving various organs are associated with SCD, such as selenic sequestration, hepatic sequestration, acute kidney injury, stroke, pulmonary hypertension, thromboembolic phenomenon, and many more^1^,^4^,^5^. The presence of HbS induces the formation of sticky patches and polymerization of HbS to bring about a significant structural alteration of red blood cells (RBC) to sickle cell shape. Due to the structural alteration, the RBCs become rigid and interferes with their interaction with other cells like platelets, leucocytes, monocytes, and endothelium, leading to vaso-occlusion of the small capillaries and hemolysis of red cells. Secondary to the repeated episodes of microvascular occlusion, the tissues are exposed to ischemia-reperfusion injuries and initiate an inflammatory response. The cellular response includes the activation of neutrophils, lymphocytes, monocytes, endothelial cells, natural killer (NK) cells, and other immune-related cells and releasing mediators for the inflammatory response^6^–^8^. The altered immune-inflammatory response relates to morbidity in SCD cases, and studies are required to uncover the underlying interplay of cytokines in the pathophysiology of the disease severity.

Our previous study report on altered T-cell response suggested failure to activate the naïve T cell to effector cells and thus have impaired immune activation in SCD cases^9^. However, immune dysregulation is brought due to the various cytokines such as tumor necrosis factor-α (TNFα), interferon-γ (IFNγ), interleukin-10 (IL10), transforming growth factor-β (TGFβ), IL6 and many others, that mediate the inflammatory and anti-inflammatory responses. IL10 is known for its anti-inflammatory role but elevated IL10 in SCD cases has been linked to interference with antigen presentation and T-cell activation^6^,^8^. Similarly, elevated TNFα, and IFNγ in individuals with SCD have been associated with disease crisis events^6^,^10^–^12^. These mediators activate an inflammatory cascade and create oxidative stress, which is highly destructive to the red cells. A compromised antioxidant defense system would further make these cells vulnerable. Glutathione is the principal protector for the RBCs; hence, its adequacy would be critical for a hemolytic crisis^13^. Various studies have demonstrated the immune-inflammatory pattern in SCD, yet the results are inconsistent and inconclusive to suggest a specific group of mediators as pro-inflammatory or anti-inflammatory. Further studies are needed to fill up the knowledge gap.

Thus, the present study focused on evaluating the serum cytokines in SCD cases and understanding the interplay of these mediators with immune cells and their impact on disease severity. The study report would enable the readers to understand the immune-inflammatory basis of the clinical course in sickle cell.

## Methods

### Ethics statement

The institute research cell and ethics committee reviewed and approved the present study. The study was performed following the ethical standard described in an appropriate version of the 1975 Declaration of Helsinki, as revised in 2000. Written informed consent was taken from all participants/their legal representatives before enrolment for the study.

### Subjects and samples

The comparative study included fifty-eight HbSS-confirmed SCD cases and thirty healthy individuals as the control group. After enrolment, the demographic and clinical profiles were entered per the approved format. As per the clinical presentations, the cases were assigned a score based on the severity scoring system proposed by Adegoke and Kuti and validated by Alabid et al.^14^–^16^. Presence of any sign and symptoms were considered assigned a score one. The patients were grouped as per the disease severity score (DS-score) more than 5 (DSscore>5) and less than equal to 5 (DSscore≤5). Individuals with a recent history of blood transfusion within three months, those diagnosed with autoimmune disorders, and undergoing immunotherapy, were excluded from the study. Blood samplewere collected in tubes containing clot activator and others containing ethylene-diamine-tetraacetic acid (EDTA). Serum was separated from the clot activator tube and analyzed for IL10, TNFα, IFNγ, and total glutathione by enzyme-linked immunosorbent assay (ELISA). The whole blood in the EDTA tube was analyzed for T cell markers by flowcytometry using DuraClone IM T cell from Beckman Coulter. Presence of markers CD45RA+197+ were considered naïve T cells while CD45RA+197-were considered effector cells. The CD279+ represented the exhausted cells, CD57+ as the senescent cells and CD27-28- as the late effector memory T cells re-expressing (TEMRA) cells. Accordingly a ratios were calculated for Th to Tc (CD4+:CD8+), naïve to effector T cell (NECR), exhausted to TEMRA cell ratio (ETCR), exhausted to effector cell ratio (EECR) and TEMRA to effector cell ratio (TECR) were calculated for Th (CD4+), Tc (CD8+), and total (T) that included both Th and Tc.

### Statistical analysis

The software IBM@SPSS version 26 was used for the data analysis for the study. The quantitative parameters were checked for normality distribution. Accordingly, analysis of variance (ANOVA) with Tukey's test was applied to compare the parameters (normally distributed) among the three groups. For the matters not showing a normal distribution curve, the parameters were compared using a Kruskal-Wallis one-way analysis of variance. Pearson correlation was performed to understand the linear association between the parameters. We performed a linear and multivariate regression model to identify the marker that significantly influenced the DSscore. The correlation network was created using Cytoscape version 3.1.0. The thickness of the lines connecting the nodes of the parameters represented the strength and the type of association^6^,^17^. A p-value<0.5 was considered statistically significant.

## Results

The study population of eighty-eight participants comprised twenty-one SCD cases with DSscore>5 and thirty-seven SCD cases with DSscore≤5 and thirty control group. The age group ranged from 7 – 45 years. A comparison of the quantitative variables between the groups is shown in Table 1. The mean (SD) Hb in SCD cases was 8.82 (1.9) gm/dL, which was significantly lower in the cases than in the control group (p<0.001). Hb in cases with DSscore>5 had grossly reduced Hb than those with score≤5 (p=0.001). The Hct and RBC count was significantly lower in cases, whereas the HbF, TLC, and Lym values were considerably higher in cases than in the control group.

**Table 1:** Comparison of parameters in three groups (n=88)

	DSscore >5 (N=21)^A^			DSscore ≤5 (N=37)^B^			Control (n=30)^C^					
	Mean (SD)	Median (IQR)	Range	Mean (SD)	Median (IQR)	Range	Mean (SD)	Median (IQR)	Range	p-value^A vs B^	p-value^A vs C^	p-value^B vs C^
***Hematological Parameters***												
Hb (g/dL)	7.5 (1.9)	7.7 (2.95)	4.20-11.20	9.56 (1.4)	9.20 (2.1)	6.50-12.4	14.19 (1.98)	14.4 (2.3)	10.4-17.7	<0.001^a^	<0.001^a^	<0.001^a^
Hct (%)	22.53 (5.3)	21.7 (8.3)	13.30-33.40	29.05 (4.6)	28.2 (7.8)	20.40-41.7	43.42 (5.35)	44.5 (5.7)	32.3-53.5	<0.001^a^	<0.001^a^	<0.001^a^
RBC count (x10^6^/µL)	3.54 (4.2)	2.54 (0.85)	1.14-21.70	3.55 (0.74)	3.52 (1.01)	2.19-5.24	4.88 (0.51)	4.94 (0.86)	3.79-5.83	0.99^a^	0.073^a^	0.033^a^
HbF (%)	14.85 (8.5)	15.5 (14.7)	0.8-28.6	15.78 (8.5)	16.7 (15)	0.8-28.7	0.84 (0.12)	0.8 (0.1)	0.6-1.1	0.87^a^	<0.001^a^	<0.001^a^
TLC (x10^3^/µL)	12.65 (6.08)	11.23 (10.72)	4.89-24.93	8.12 (3.96)	6.90 (4.72)	3.82-23.57	6.96 (1.1)	7.01 (1.6)	4.09-8.9	<0.001^a^	<0.001^a^	0.465^a^
Lymphocytes (cells/mm^3^)	3281.61 (1514.7)	3417.75 (2410.5)	535.44-6185.68	1810.97 (957.23)	1547.07 (1358.18)	384.16-3786.24	1619.73 (499.95)	1583.38 (652.37)	821.1-2763.93	<0.001^a^	<0.001^a^	0.72^a^
***Serum markers***												
TNFα (pg/mL)	152.04 (181.93)	100.51 (66.8)	2.24-770.95	76.12 (79.9)	45.63 (75.33)	0.58-390.83	99.97 (158.85)	45.9 (88.01)	2.43- 800	0.04^b^	0.073^b^	0.91^b^
IFNγ (pg/mL)	49.15 (52.15)	44.60 (70.24)	0.36-192.59	54.08 (73.31)	20.20 (63.24)	0.45-257.15	14.05 (34.14)	1.33 (4.24)	0.3-138.17	0.95^b^	0.01^b^	0.002^b^
IL10 (pg/mL)	11.44 (10.8)	7.9 (13.3)	0.09-39.11	2.06 (1.95)	1.31 (2.73)	0.02-7.13	0.89 (1.3)	0.5 (0.53)	0.03-6.98	0.001^b^	<0.001^b^	0.02^b^
Glutathione (µM)	2.97 (4.1)	2.18 (1.53)	0.45-20.32	1.65 (0.71)	1.50 (0.62)	0.41-4.24	1.76 (0.73)	1.62 (1.15)	0.73-3.43	0.057^b^	0.106^b^	0.98^b^
***T-Lymphocyte markers***												
CD3+ (cells/mm^3^)	2087.98 (1045.9)	2072.32 (1484.5)	296.63-4447.50	1181.5 (598.84)	1194.34 (860.05)	217.24-2575.07	1203.15 (414.84)	1138.26 (613.54)	496.84-2372.63	<0.001^a^	<0.001^a^	0.99^a^
Th cells (CD4^+^) (cells/mm^3^)	653.46 (409.18)	601.28 (477.83)	134.38-1867.95	433.68 (235.36)	387.48 (286.83)	53.66-924.62	439.13 (168.91)	432.78 (286.74)	164.95-735.51	0.01^a^	0.017^a^	0.99^a^
Tc cells (CD8^+^) (cells/mm^3^)	538.1 (475.7)	359.91 (527.77)	37.67-2160.86	268.59 (168.8)	260.56 (249.6)	44.97-836.89	346.3 (191.3)	307.34 (238.01)	91.92-1043.96	0.002^a^	0.046^a^	0.49^a^
CD4^+^:CD8^+^	1.88 (1.12)	1.84 (1.65)	0.13-4.03	1.9 (1.2)	1.53 (1.14)	0.68-7.47	1.46 (0.6)	1.37 (1.11)	0.57-2.46	0.99^a^	0.32^a^	0.19^a^
Immunosenescent cells Th cells (CD4^+^57^+^) (cells/mm^3^)	43.26 (46.8)	27.14 (58.02)	1.87-184.93	12.47 (11.25)	8.86 (18.9)	0.43-38.17	25.67 (41.96)	15.03 (24.43)	1.54-229.34	0.006^b^	0.4^b^	0.28^b^
Exhausted Th cells (CD4^+^279^+^) (cells/mm^3^)	179.74 (180.06)	116.38 (184.35)	24.19-827.50	68.02 (42.05)	59.81 (69.78)	5.85-163.66	70.37 (41.5)	61.89 (61.9)	19.03-175.8	0.005^b^	0.013^b^	0.99^b^
Immunosenescent cells Tc cells (CD8^+^57^+^) (cells/mm^3^)	225.49 (244.6)	147.42 (251.97)	12.14-944.30	100.99 (117.65)	63.45 (105.22)	7.90-679.56	137.55 (141.08)	106.96 (126.3)	11.21-762.09	0.018^b^	0.15^b^	0.64^b^
Exhausted Tc cells (CD8^+^279^+^) (cells/mm^3^)	210.75 (224.6)	124.27 (200.23)	7.53-1006.96	77.63 (50.18)	66.92 (67.62)	10.70-211.34	107.62 (65.22)	87.82 (86.62)	29.2-268.6	<0.001^b^	0.009^b^	0.57^b^
CD4^+^ Th Naïve/Effector cell ratio	44.13 (40.13)	29.4 (54.9)	3.32-147.25	91.24 (107.95)	60.7 (113.6)	1.81-456.0	52.32 (67.85)	32.32 (55.84)	0.07-317.0	0.1^b^	0.94^b^	0.14^b^
CD4^+^ Th Exhausted/TEMRA cell ratio	28.26 (52.14)	8.27 (30.18)	0.72-233.0	29.71 (70.17)	7.88 (26.8)	0.17-413.0	10.01 (12.59)	4.34 (11.4)	0.66-50.5	0.99^b^	0.45^b^	0.29^b^
CD4^+^ Th Exhausted/Effector cell ratio	49.29 (65.12)	25.6 (57.4)	0.79-243.0	51.13 (65.75)	23.64 (65.56)	0.55-247.0	20.79 (21.7)	10.92 (33.6)	0.88-72.0	0.99^b^	0.165^b^	0.067^b^
CD4^+^ Th TEMRA/Effector cell ratio	4.77 (7.72)	2.4 (3.9)	0.17-36.14	4.75 (9.82)	2.0 (3.34)	0.33-60.0	3.31 (4.1)	1.9 (2.6)	0.09-22.0	0.98^b^	0.79^b^	0.73^b^
CD8^+^ Tc Naïve/Effector cell ratio	1.99 (2.18)	1.17 (2.77)	0.15-8.5	2.22 (2.2)	1.7 (1.95)	0.18-9.69	2.51 (2.8)	1.51 (2.5)	0.19-11.77	0.94^b^	0.72^b^	0.87^b^
CD8^+^ Tc Exhausted/TEMRA cell ratio	2.11 (3.18)	1.28 (1.32)	0.34-15.19	1.81 (2.6)	0.96 (1.59)	0.27-15.87	2.09 (1.9)	1.19 (2.03)	0.29-7.16	0.9^b^	0.9^b^	0.89^b^
CD8^+^ Tc Exhausted/Effector cell ratio	2.12 (1.95)	1.69 (1.28)	0.39-7.6	1.39 (0.8)	1.23 (1.37)	0.26-3.08	1.87 (1.46)	1.23 (2.2)	0.34-5.95	0.14^b^	0.81^b^	0.33^b^
CD8^+^ Tc TEMRA/Effector cell ratio	1.29 (0.61)	1.19 (0.55)	0.5-3.4	1.1 (0.43)	1.06 (0.51)	0.19-2.35	1.06 (0.48)	1.004 (0.53)	0.47-2.88	0.303^b^	0.21^b^	0.94^b^
Total Naïve/Effector T cell ratio	5.11 (5.97)	2.76 (5.77)	0.39-23.17	4.57 (3.68)	4.002 (4.03)	0.32-17.72	4.69 (4.32)	3.09 (5.25)	0.52-17.58	0.89^b^	0.94^b^	0.99^b^
Total Exhausted/TEMRA T cell ratio	3.52 (5.7)	2.2 (2.2)	0.37-27.4	2.71 (2.9)	1.42 (2.9)	0.26-12.86	2.55 (2.3)	1.49 (1.56)	0.42-8.88	0.69^b^	0.61^b^	0.98^b^
Total Exhausted/Effector T cell ratio	4.22 (4.03)	3.2 (3.35)	0.46-17.7	2.57 (1.96)	1.79 (2.76)	0.4-8.75	2.5 (1.65)	2.18 (2.46)	0.44-6.75	0.049^b^	0.049^b^	0.99^b^
Total TEMRA/Effector T cell ratio	278.98 (449.68)	153.14 (223.73)	9.69-2069.57	137.94 (140.1)	100.24 (114.39)	14.83-652.1	118.79 (113.8)	89.84 (101.26)	14.68-617.19	0.095^b^	0.062^b^	0.95^b^

a: One way ANOVA test

b Kruskal-Wallis test

p<0.05 considered as statistically significant

Hb:hemoglobin, Hct:hematocrit, RBC:red blood cell, TLC:total leucocyte count, HbF:fetal hemoglobin, Lym:absolute lymphocyte count, CD3^+^:absolute T lymphocyte, CD4^+^:helper T cells, CD8^+^:cytotoxic T cells, CD4^+^: CD8^+^:CD4^+^ to CD8^+^ T cell ratio, CD4^+^57^+^:immunosenescent helper T cells, CD4 ^+^279^+^:exhausted helper T cells, CD8 ^+^57^+^:immunosenescent cytotoxic T cells, CD8 ^+^279^+^:exhausted cytotoxic T cells, NECR:naïve to effector T cell ratio, ETCR:exhausted to TEMRA T cell ratio, EECR:exhausted to effector T cell ratio, TECR:TEMRA to effector T cell ratio, TEMRA: Late effector memory T cell re-expressing, T-NECR/ETCR/EECR/TECR:total T cells including both CD4^+^ and CD8^+^ T cells, TNFα-tumor necrosis factor-α, IFNγ-interferon-γ, IL10:interleukin-10, Glut:glutathione

The serum TNFα level was higher in cases (p=0.35) and ranged from 0.58-770.95 pg/mL with a median (IQR) of 77.32 (80.91) pg/mL. The cases with DSscore>5 delineated a higher TNFα level than those with lesser score (p=0.04, Table 1). In the cases, the median (IQR) of serum IFNγ, IL10, and glutathione levels were 28.53 (64.12) pg/mL, 2.1 (4.88) pg/mL, and 1.56 (0.93) µM, respectively. The respective ranges in the cases were 0.36- 257.15 pg/mL, 0.02- 39.11 pg/mL, and 0.41- 20.32 µM. Compared to the control group, the median IFNγ and IL10 levels were significantly higher in both the groups of cases (Table 1). The IL10 levels were grossly elevated in patients with DSscore>5 (p=0.001) than the other two. Serum glutathione was nearly similar in all three groups (p=0.088).

The mean (SD) CD3+ T lymphocyte count in cases with DSscore>5 and ≤5 was 2087.98 (1045.9) and 1181.5 (598.84) cells/mm3, respectively (Table 1). The count was greatly elevated in the cases with DSscore>5 than those with a score≤5 and the control group (p=0.001). Patients with higher DSscore reported raised Th and Tc cell counts than those with lower scores (p=0.01 & 0.002) and the control group (p=0.017 & 0.046). The mean (SD) CD4+ Th cells in DSscore >5, ≤5 and the control group were 653.46 (409.18), 433.68 (235.36), and 53.66-924.62 cells/mm^3^, respectively. Similarly, the CD8+ Tc cell counts were 538.1 (475.7), 268.59 (168.8), and 346.3 (191.3) cells/mm3. The counts were equivocal in cases with DSscore ≤5 and the control group, as was the ratio for CD4+/CD8+cells (p>0.05).

Absolute counts of immunosenescent (CD57+) CD4+ Th and CD8+ Tc cells were found to be higher in cases with DSscore>5 than those with lower crisis score values (p=0.004 & 0.018). Similarly, the cases with higher score revealed an increased count of exhausted (CD279+) CD4+ Th and CD8+ Tc cells (p<0.001). To understand the overall process of immune activation, ratios were calculated. Te NECR provided an idea for the number of naïve cell being activated to effector cells and predict the iimune reconstitution^18^.

Similarly, the number of exhausted cell over effector cells (EECR) marks for excessive immune response leading to T cell hyporesponsiveness^19^,^20^. The ratio of exhausted to TEMRA cells (ETCR) indicate the T cell with terminally differentiated property and provided an insight regading control on excessive effector response^21^,^22^. TECR represented cells that have lost proliferative capacity but still have cytotoxic activity and an ability to develop memory response dring the ongoing immune response^23^.

**Figure 1:**
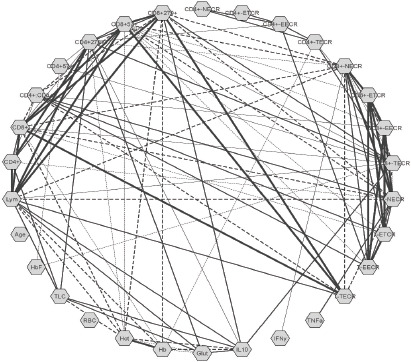
Correlation analysis presented as network of the quantitative parameters analysed in the cases (n=58) Note: Each parameter presented as a node. Statistical analysis computed using the Cytoscape and p<0.05 was considered significant by the lines connected to both nodes. The continuous lines represented the positive correlation and the dashed lines represen ted the negative correlation. The strength of the correlation, -value, represented by the line thickness: strong (r>0.68), moderate(0.36≥r≥0.68), and weak (r<0.36). Hb:hemoglobin, Hct:hematocrit, RBC:red blood cell, TLC:total leucocyte count, HbF:fetal hemoglobin, Lym:absolute lymphocyte count, CD4 ^+^:helper T cells, CD8 ^+^:cytotoxic T cells, CD4^+^: CD8^+^:CD4^+^ to CD8 ^+^ T cell ratio, CD4^+^57^+^:immunosenescent helper T cells, CD4 ^+^279^+^:exhausted helper T cells, CD8 ^+^57^+^:immunosenescent cytotoxic T cells, CD8^+^279^+^:exhausted cytotoxic T cells, NECR:naïve to effector T cell ratio, ETCR:exhaustedto TEMRA T cell ratio, EECR:exhausted to effector T cell ratio, TECR:TEMRA to effector T cell ratio, TEMRA: Late effector memory T cell re-expressing, T-NECR/ETCR/EECR/TECR:total T cells including both CD4 ^+^ and CD8^+^ T cells, TNF a-tumor necrosis factor-α, IFNγ-interferon-γ, IL10:interleukin-10, Glut:glutathione

The correlation network of the quantitative parameters following multivariate analysis in SCD cases is illustrated in Figure 1. Serum IL10 depicted a significant inverse association with Hb (r= -0.4, p=0.002) and Hct (r= -0.44, p=0.001). It revealed a significant positive correlation with absolute lymphocyte count (r= 0.412, p=0.001), CD3+T cells (r=0.324, p=0.013), Tc exhausted (CD8+CD279+) cells (r= 0.327, p=0.012), and Th exhausted (CD4+CD279+) cell (r=0.265, p=0.045). Serum IL10 recorded a significant linear relationship with Tc TEMRA/Effector cell ratio (CD8+TECR) (r=0.337, p=0.01). A linear association (r=0.292, p=0.026) was observed between IFNγ and Tc Naive/Effector cell ratio (CD8+NECR).

Similarly, TNFα was positively associated with the total T cell Naive/Effector cell ratio (TNECR) (r=0.307, p=0.019). Serum glutathione levels correlated significantly with absolute lymphocyte count (r=0.31, p=0.018), Th exhausted (CD4+CD279+) cell (r=0.393, p=0.002) and IL10 (r=0.589, p<0.001). It also revealed an inverse relationship with Hb (r= -0.261, p=0.048) and Hct (r= -0.275, p=0.036). It was observed that CD8+Tc cells, especially the exhausted CD8+Tc (CD279+) cells depicted a negative correlation with Hb (r= -0.39, p=0.003) and Hct (r= -0.4, p=0.002). Overall in cases, Hct was found to be affected by raised Tc (p=0.29) and Th (0.07) immunosenescent (CD57+) cell counts.

**Figure 2:**
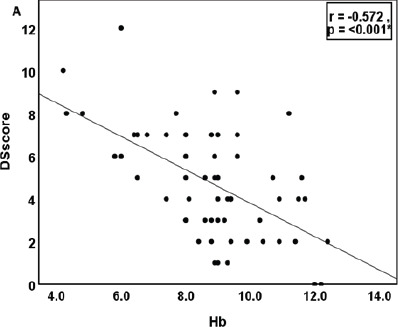

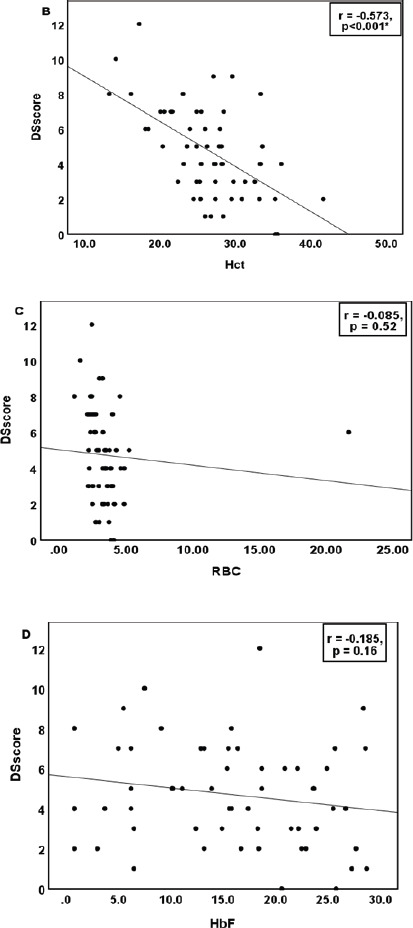

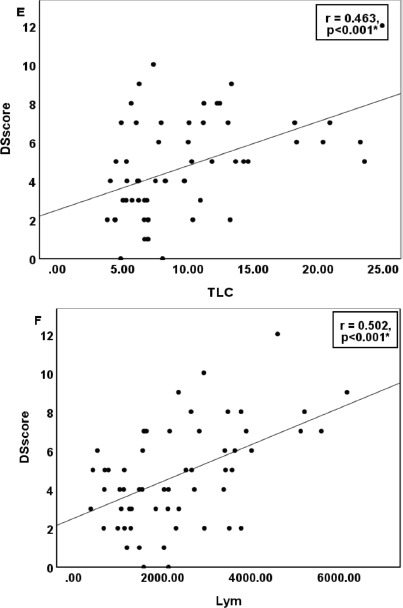
Correlation of hematological parameters with Disease Severity Score in cases (n 58) Note: p<0-05 considered as statistically significant. DSscore: disease severity score, Hb:hemoglobin, Hct:hematocrit, RBC:red blood cell, TLC:total leucocyte count, HbF:fetal hemoglobin, Lym:absolute lymphocyte count

**Figure 3:**
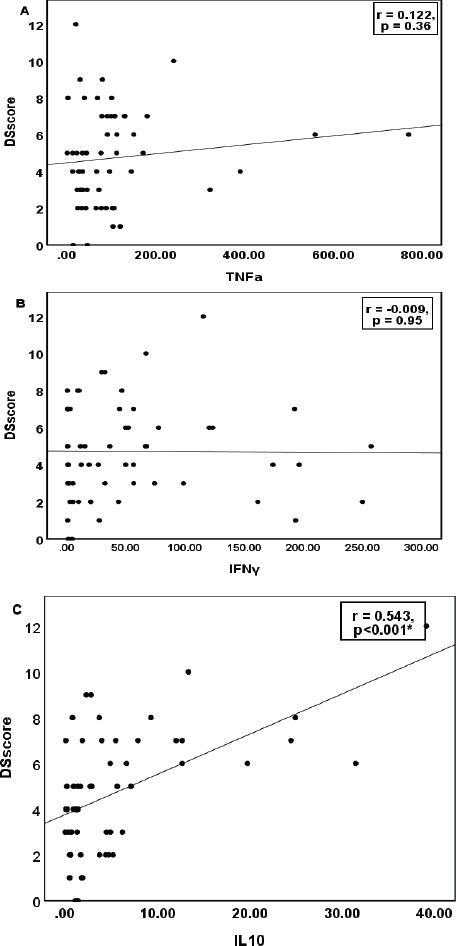

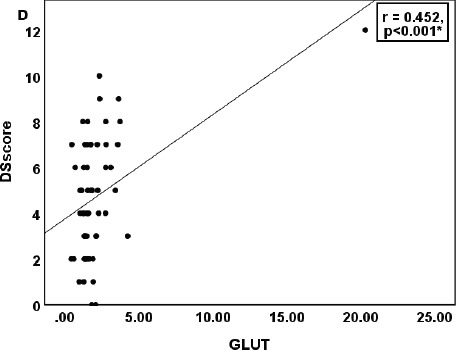
Correlation of serum inflammatory and anti-inflammatory markers with Disease Severity Score in cases (n=58) Note: p<0.05 considered as statistically significant. DSscore: disease severity score, TNFα-tumor necrosis factor-α, IFNγ-interferon-γ, IL10:interleukin-10, GLUT:glutathione

**Figure 4:**
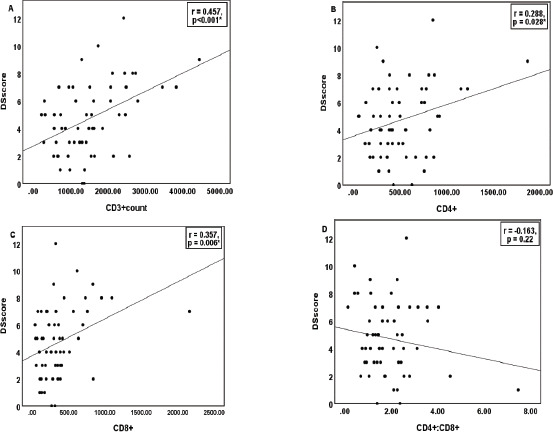

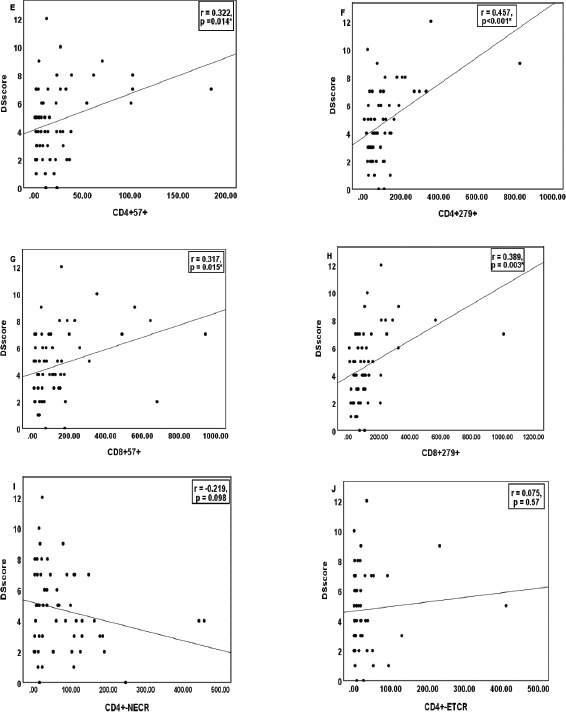

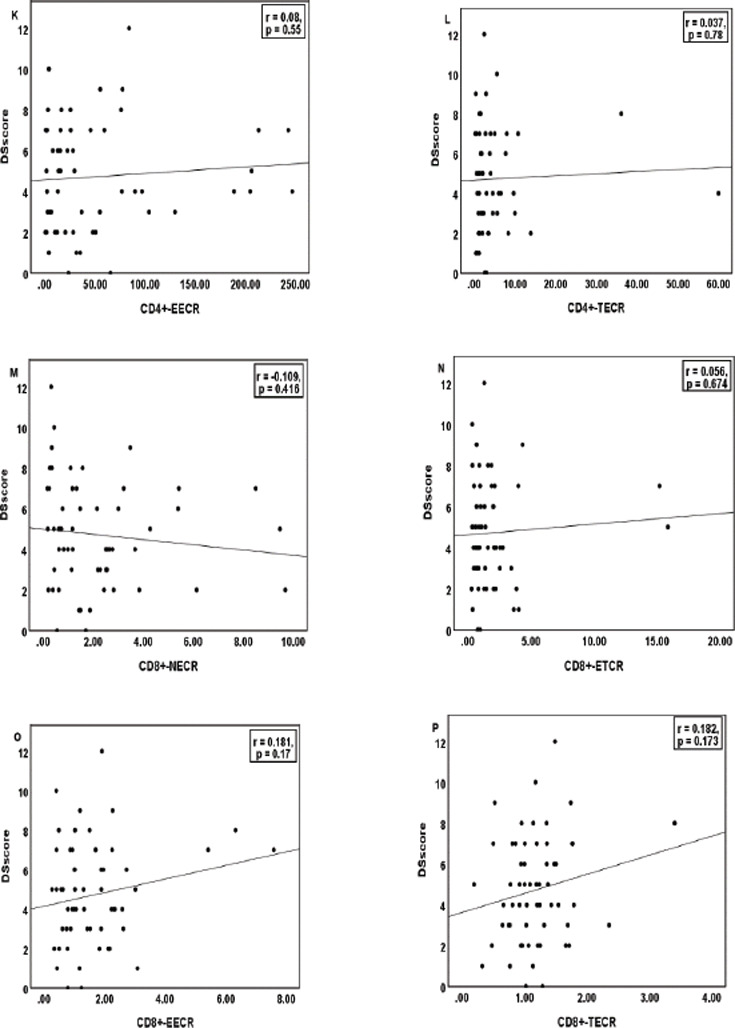

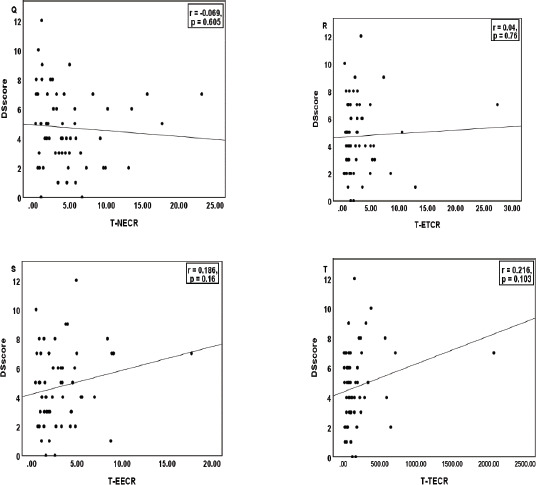
Correlation of T-cell markers with Disease Severity Score in cases (n=58) Note: p<0.05 considered as statistically significant. DSscore: disease severity score, CD3^+^:absolute T lymphocyte, CD4^+^:helper T cells CD8^+^:cytotoxic T cells, CD4^+^: CD8^+^:CD4^+^ to CD8^+^ T cell ratio, CD4^+^57^+^:immunosenescent helper T cells, CD4^+^279^+^:exhausted helper T cells, CD8^+^57^+^:immunosenescent cytotoxic T cells, CD8 ^+^279^+^:exhausted cytotoxic T cells, NECR:naïve to effector T cell ratio, ETCR:exhausted to TEMRA T cell ratio, EECR:exhausted to effector T cell ratio, TECR:TEMRA to effector T cell ratio, TEMRA: Late effector memoiy T cell re-expressing, T-NECR/ETCR/EECR/TECR:total T cells including both CD4^+^ and CD8^+^ T cells

The correlation of the quantitative parameters with DSscore is depicted in Figure 2-4. Hb and Hct showed a significant negative correlation, while TLC and absolute lymphocyte count showed a positive correlation (Figure 2). Serum IL10 and glutathione recorded a strong positive association with DSscore (p<0.001), as shown in Figure 3. Similarly, CD3+, CD4+Th, and CD8+Tc cells correlated linearly with DSscore (Figure 4A-C). A similar association was also observed for the immunosenescent cells (57+) and exhausted cells (279+) CD4+Th and CD8+Tc cells (Figure 4E-H).

## Discussion

SCD is associated with microvascular obstruction due to enhanced adhesiveness of the rigid sickled erythrocytes and reticulocytes. The repeated insults due to subclinical microinfarctions create a state of chronic inflammation in the individual with the disease. The immune-inflammatory response initiates the activation of lymphocytes and other immune cells involved in this clinical course. Consequent to the activation, a group of inflammatory and anti-inflammatory cytokines such as TNFα, IFNγ, IL10, and other biomolecules are released to regulate the process 4,6,7. Very limited studies have evaluated the interaction of these markers and their association with the clinical impact, especially crisis events in SCD patients. The previous study report on the derangement of the various T-cell subsets suggested immune inactivation in these patients with SCD. Elevated naïve Th and Tc cells were accompanied by decreased effector memory and central memory T cells in crisis state than the steady state group (p<0.05)^9^. The present finding provides insight into the interplay of cytokines with immune cells and their impact on disease severity. The study report would enable the readers to understand the immune-inflammatory basis of the clinical course.

Compared to the healthy control group, not only did the cases with higher DSscore (≥5) depict grossly elevated inflammatory markers, TNFα, IFNγ, and IL10 (Table 1), even the cases with less DSscore (<5) reported a similar result.

These biomolecules contribute to the clinical manifestations evidenced by the patients during the crisis episodes. Elevated levels of inflammatory cytokines justified a state of chronic inflammation in these patients that might be due to an altered immune response. Serum IL10 has been associated with T cell differentiation, vascular crisis events, and clinical severity, suggesting a potential role of this cytokine in regulating the immune-inflammatory cascade^24^. Study reports have indicated that various group of cells like this pleiotropic mediator is produced by T-helper type 2 (Th2) cells, regulatory T (Treg) cells, Th1 effector cells, dendritic cells, NK cells, activated monocytes/macrophages, damaged endothelium produce IL10 that regulates an exaggerated inflammatory reaction and thus exerts an anti-inflammatory effect. It prevents lymphocyte activation by the antigen-presenting cells (APCs) and inhibits the release of pro-inflammatory cytokines^6^,^11^,^25^. However, thenti-inflammatory property of IL10 might be critical in SCD patients, which might reduce the number of effector cells for an adequate immune response to tackle the clinical worsening. Various uncharacterized signaling pathways were reported to modulate the inflammatory and anti-inflammatory effects of IL10 in SCD but with conflicting conclusions. Garcia et al. study observed grossly increased IL10 in SCA patients at a steady state than the healthy control group (p<0.001) 17. The remarkable rise of serum IL10 was seen in SCA cases with high death risk than those with a lower death risk. The study demonstrated immunological crosstalk indicating CD4^+^ T cells, VLA-4 in CD8+ T cells, Th1, Th17 as inflammatory, and IL10 as regulatory cytokines to control endothelial adhesion (17). Viega et al. showed an increase in IL10 levels in the SCA group than in the control group (p<0.05) 10.

On the contrary, Sarray et al. reported reduced serum IL10 levels and raised IL6 in SCD cases with VOC compared to the steady state group 11. The study supported a strong association between IL10 and IL6, and the reduced anti-inflammatory capacity of IL10 might be a potential cause for inflammation in crisis 11. Siransy et al. suggested the anti-inflammatory effect of IL10 and IL33 26. The study noted decreased serum levels of IL10 (p=0.1) and IL33 (p=0.04) in the crisis state than the steady state. The inflammatory cytokines IL6, IL1β, IL18, and TNFα were raised in the crisis group. However, IL10 was found elevated in SCD cases with VOC compared to those presenting with hemolytic anemia^26^. The study findings indicated an integrated response of various cytokines in clinical exacerbation. Activated leucocytes, platelets, and endothelial cells stimulate the production of a cascade of cytokines characterizing the clinical manifestation during the inflammatory response. In the present study, the immunosenescent and exhausted T (CD4+ and CD8+) cells and IL10 were profoundly elevated in cases with DSscore ≥5 than those with lower DSscore (Table 1). The linear rise in serum IL10 levels with DSscore (p<0.001, fig.-3c) in the cases reflect the compensatory counter-regulatory mechanism to the ongoing inflammatory cascade. Consequent to the repeated ischemic insults to the tissues, a consistent inflammatory state is set that need to be compensated. Activation of anti-inflammatory system is a very natural host response to stress. However, the immune cells and cytokines can lead both ways, protective or pathogenic, depending upon the interaction with other cytokines^24^.

As a result of the immune-inflammatory cascade, the activated monocytes, the sickled-shaped erythrocytes with their adhesion molecules, activated leucocytes, especially the polymorphonuclear leucocytes, release inflammatory mediators, like TNFα, IL-1β, IL6, and IFNγ. The cytokines sustain a forwarding cascade effect on the leucocytes, endothelial cells, and platelets triggering vaso-occlusive events^11^,^17^,^21^. Besides, these mediators have been implicated in T cell differentiation and expansion in SCD 10. Serum TNFα level was higher in patients with DSscore ≥5 than those with <5 (p=0.04, Table 1); however, IFNγ levels were nearly smilar. Pathare et al. observed an elevated TNFα in SCD cases with crisis than the steady state cases and the healthy control group (p>0.05)^27^. The IFNγ levels were higher in steady state cases compared to the control group (p=0.02). The study implied that SCD patients have a persistent elevation of pro-inflammatory cytokines, IL1β, and IFNγ, with an augmentation of cytokine TNFα during crisis events^27^. Selvaraj et al. study depicted increased TNFα gene expression in monocytes of SCD patients than those from the healthy control group 28. Similarly, Bandeira et al. study documented an increased inflammatory profile in SCA cases compared to healthy control suggesting an association with the genetic characteristics^12^. Serum IL6 and TNFα were raised in SCA with the Bantu/Bantu haplotype than the Benin/Benin haplotype, whereas the Bantu/Benin haplotype depicted reduced levels^12^. On the contrary, Garcia et al. reported no significant differences in serum TNFα levels in sickle cell anemia (SCA) and the control group 17. The present study observed an increase in TNFα and IFNγ in cases with higher DSscore than those with lower scores and the healthy control group (Table 1) but failed to depict any significant association with DSscore (fig.3a & b). The Th1 cytokines, TNFα, IFNγ, and IL2 mainly regulate the cell-mediated inflammatory responses, including macrophage activation, complement-fixing, and antibody dependant cell-mediated cytotoxicity^27^. A significant positive association of TNFα and IFNγ with an elevated naïve cell over the effector cell (NECR, fig.1) supported immune inactivation in the present study. A complex interplay of these cytokines with the T cells, failed to activate the naïve T cell to their effector cells could be due to the presence of some inhibitory factors^7^,^17^.

Hydroxyurea, a ribonucleotide reductase inhibitor and a cell cycle inhibitor, could be one of such factors^9^. Similarly, oversupplementation with folic acid in SCD patients lead to accumulation of unmetabolized folic acid in the plasma, could be another inhibitory factor. Studies have reported immunosuppressive potency of unmetabolized folic acid by interfering NK cell and T regulatory cell activities^29^,^30^. On the other hand, IL10 strongly correlated with the Tc TEMRA cell count upon Effector cell count and the number of exhausted (279+) Tc cells (fig.1). The linear association emphasized the controlling effect of IL10 on further T cell activation to downregulate the inflammatory response.

Reduced glutathione is a crucial intracellular antioxidant defense to alleviate the oxidative stress triggered during hemolysis. Gizi et al. assessed the oxidant-antioxidant status in SCD^13^. The study reported decreased levels of total and reduced glutathione (p<0.001) and increased reactive oxygen species (ROS) (p<0.001) in SCD cases than the control group (13). Morris et al. study accorded significantly low total plasma glutathione level in SCD cases compared to control group (p<0.05)^31^. The study also demonstrated 61% depletion of erythrocytic glutathione in SCD cases than their control couterparts 3. As a significant endogenous intracellular antioxidant, reduced levels linearly increase oxidative stress. Together, the metabolic derangement makes the patients vulnerable to pathophysiological changes.

Although no significant association was observed between glutathione levels and disease severity, Kalejaiye et al. found significantly elevated glutathione levels in SCD cases with frequent crisis events (p=0.021) and repeated blood transfusions (p=0.049)^32^. The present study observed no significant difference in serum glutathione levels between the groups (Table 1). However, it delineated a significant positive correlation with DSscore (p<0.001, Fig.3d) might be ascribed to the compensatory increase in the endogenous antioxidative mechanism in the red cells during crisis events. As the disease severity progresses, the pathophysiology induces red cell destruction, and the hemolytic crisis prevails with the release of intracellular glutathione that justifies the negative association of glutathione with Hb and Hct (p<0.05, Fig.1). A large-scale study on the quantification of the intracellular glutathione defense system comprising the peroxidase and reductase activity would be quite insightful. Gene expression studies related to inflammatory and anti-inflammatory biomolecules might provide a more accurate understanding of the immunological response. Glutathione and IL10 are linearly correlated to each other, and both depicted a direct impact of increased counts of exhausted T cells (CD279+) in the cases (Fig.1). The rise in these biomolecules during the crisis could be a compensatory trigger to achieve a control on the quiescence and activation to bring about homeostasis.

## Limitations

The study's major limitation is the small sample size and the limited number of cytokines included in the study due to the fund constraint. One-time analysis of the biomarkers was another drawback. A prospective longitudinal follow-up of the same cohort for a set of biomarkers would provide a more precise insight into the immuno-inflammatory pathophysiology of the clinical course in SCD. However, looking into the limited number of studies on immune responses in SCD, this article would provide an essential element of understanding the underlying immune pathophysiology of the crisis event in sickle cell.

## Conclusion

The SCD cases with DSscore≤5 delineated higher IFNγ levels than the control group, indicating that a state of inflammatory activation at the subclinical level prevailed. It is associated with elevated serum levels of anti-inflammatory cytokine IL10 as a compensatory counter-regulatory mechanism. Serum TNFα was significantly increased in cases with DSscore>5, suggesting that as the disease severity increases, the immune-inflammatory cascade is augmented, as evidenced by a rise in TNFα due to release from the activated endothelium and leucocytes. The anti-inflammatory cytokine IL10 was considerably elevated in cases with DSscore >5. Serum IL10 recorded significant linear association with the exhausted T cells emphasizing its role in controlling further T cell action to downregulate the inflammatory process to achieve homeostasis. Similarly, glutathione depicted a positive correlation with DSscore explaining an activated anti-inflammatory mechanism in SCD cases during the ongoing crisis phase. Cytokine profiling in SCD patients might provide insight into the underlying clinical course and vulnerability toward crisis events.

Nevertheless, a more significant number of studies on a broad set of immune cells, and their cytokines, in the same cohort to be regularly monitored during the follow-up would provide a more precise understanding of the cytokine-immune cell signaling pathways. Gene expression analysis of these biomolecules involved in the crosstalk of immune response and inflammatory cell activation during the crisis event is needed better to understand the immune-inflammatory basis of the pathophysiology in SCD. Understanding the pathophysiology would help look forward to a targeted therapeutic approach to control the immune cell cytokine interplay and the inflammatory responses.

## Highlights

Serum cytokines, IL10, TNFα, and IFNγ, were elevated in cases compared to the control group.

SCD cases with DSscore>5 recorded raised levels of serum cytokines than those with a lower score

The immunosenescent and exhausted T cell counts were higher in cases with higher scores and depicted linear association with the cytokines and DSscore. Of all the cytokines, IL10 depicted a strong positive association with DSscore, and a negative influence on Hb and Hct, emphasizing its role as an anti-inflammatory cytokine to compensate for the ongoing inflammatory process during a crisis.

Cytokine profiling in SCD patients might provide insight into the clinical course and vulnerability toward crisis events.

## Data Availability

The data is presently available with the principal investigator (corresponding author). It is not available in the public domain due to privacy or ethical restrictions. It be made available from the corresponding author upon reasonable request. The letter shall be reviewed and approved by all the authors and the Head of the Institution, following which it shall be made available.
